# Supplementary figures and images for: Context coding in the mouse nucleus accumbens modulates motivationally relevant information
Source: PLoS Biol. 2022 Apr 29;20(4):e3001338. doi: 10.1371/journal.pbio.3001338 (PMC9094556; doi:10.1371/journal.pbio.3001338)

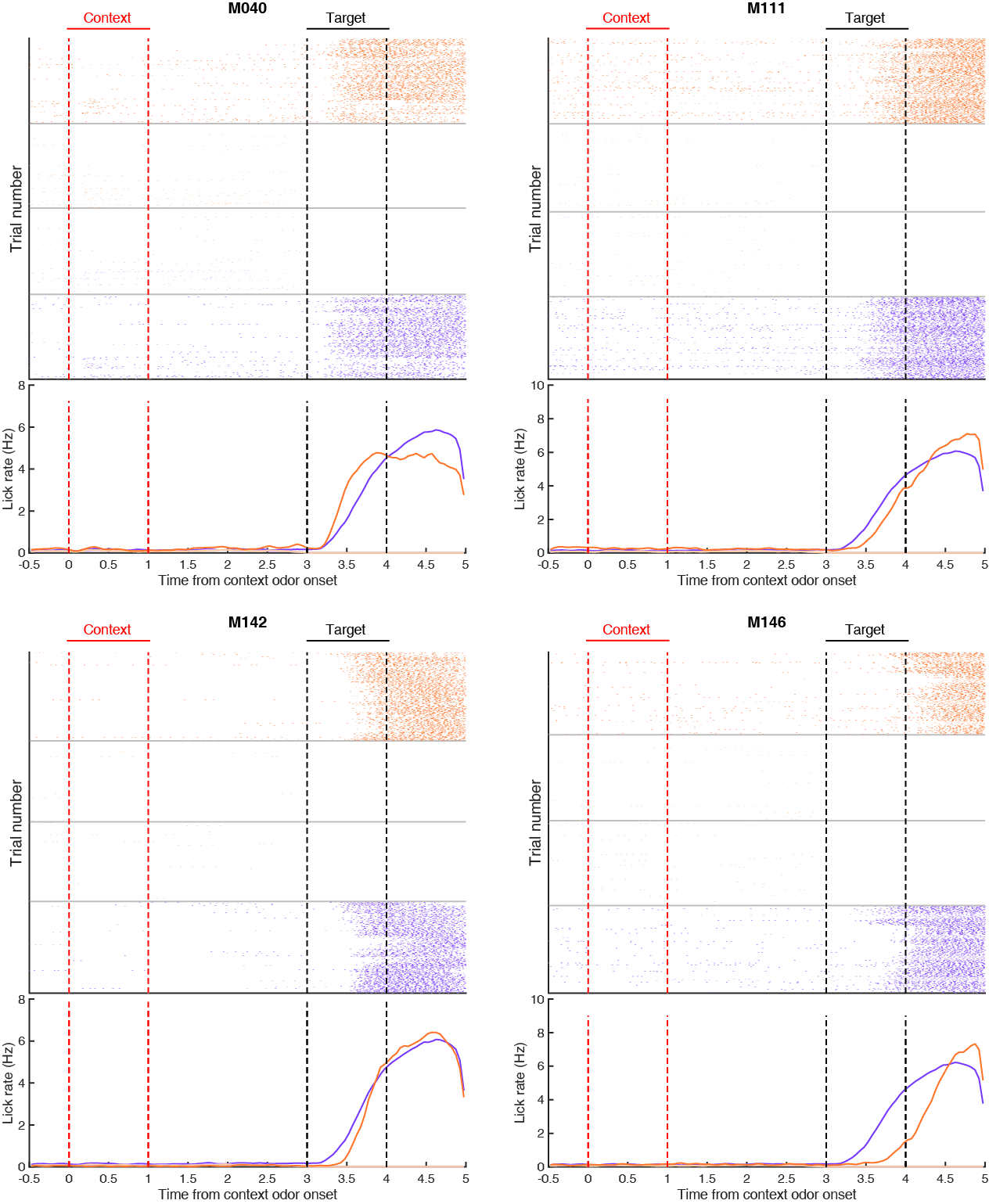

Supplement: S1 Fig — Top of each plot shows lick rasters during correct trials for the 4 trial types for all recording sessions (purple: trials with context cue O1; orange: trials with context cue O2; dark colors: rewarded trials; light colors: unrewarded trials). Bottom half of each plot shows trial-averaged licking rates for each trial type aligned to context cue onset. Data shown are averaged across all recording sessions. Context cue presentation (0–1 s) is bordered by red lines, and target cue presentation (3–4 s) is bordered by black lines. Note that for correct trials, substantial licking only appears after target cue onset, and for rewarded trials only. Data: https://gin.g-node.org/jgmaz/BiconditionalOdor. (TIF) [file pbio.3001338.s001.tif]

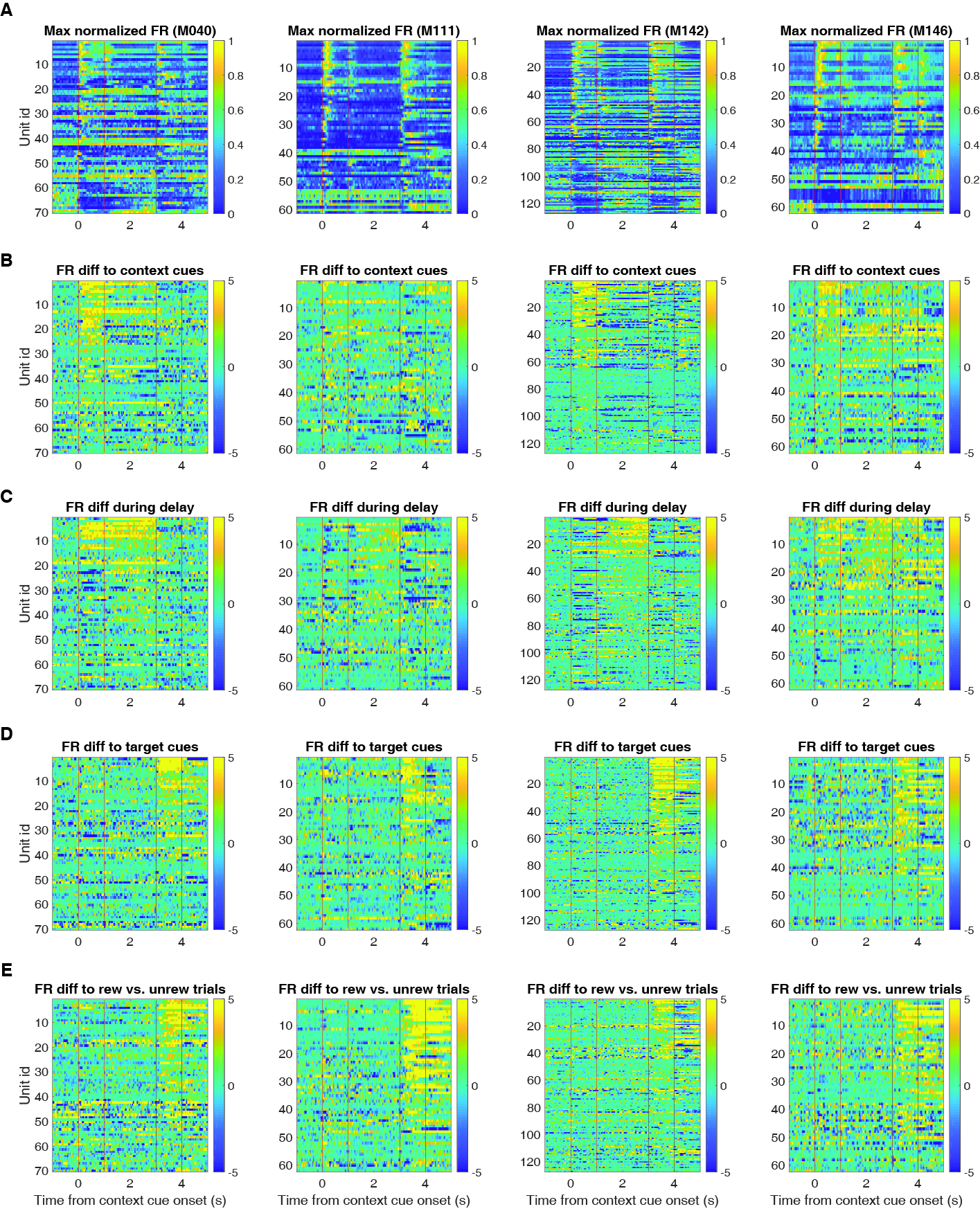

Supplement: S2 Fig — Each plot is a heat plot showing either max normalized firing rates or firing rate differences for trial-averaged data for all eligible units, with unit identity sorted according to the peak value for the comparison of interest. From left to right shows data for M040, M111, M142, and M146. Red lines border context cue presentation, and black lines border target cue presentation. (A) Firing rate profiles for units at 1-s pre- and postcontext cue onset, sorted according to maximum value after context cue onset. (B) Firing rate differences for units across context cues, sorted according to maximum difference during context cue presentation. (C) Firing rate differences for units across context cues during the delay period, sorted according to maximum difference during the 1-s period preceding target cue presentation. (D) Firing rate differences for units across target cues, sorted according to maximum difference during target cue presentation. (E) Firing rate differences for units for rewarded and unrewarded trial types during target cue presentation, sorted according to maximum difference during target cue presentation. Data: https://gin.g-node.org/jgmaz/BiconditionalOdor. (TIF) [file pbio.3001338.s002.tif]

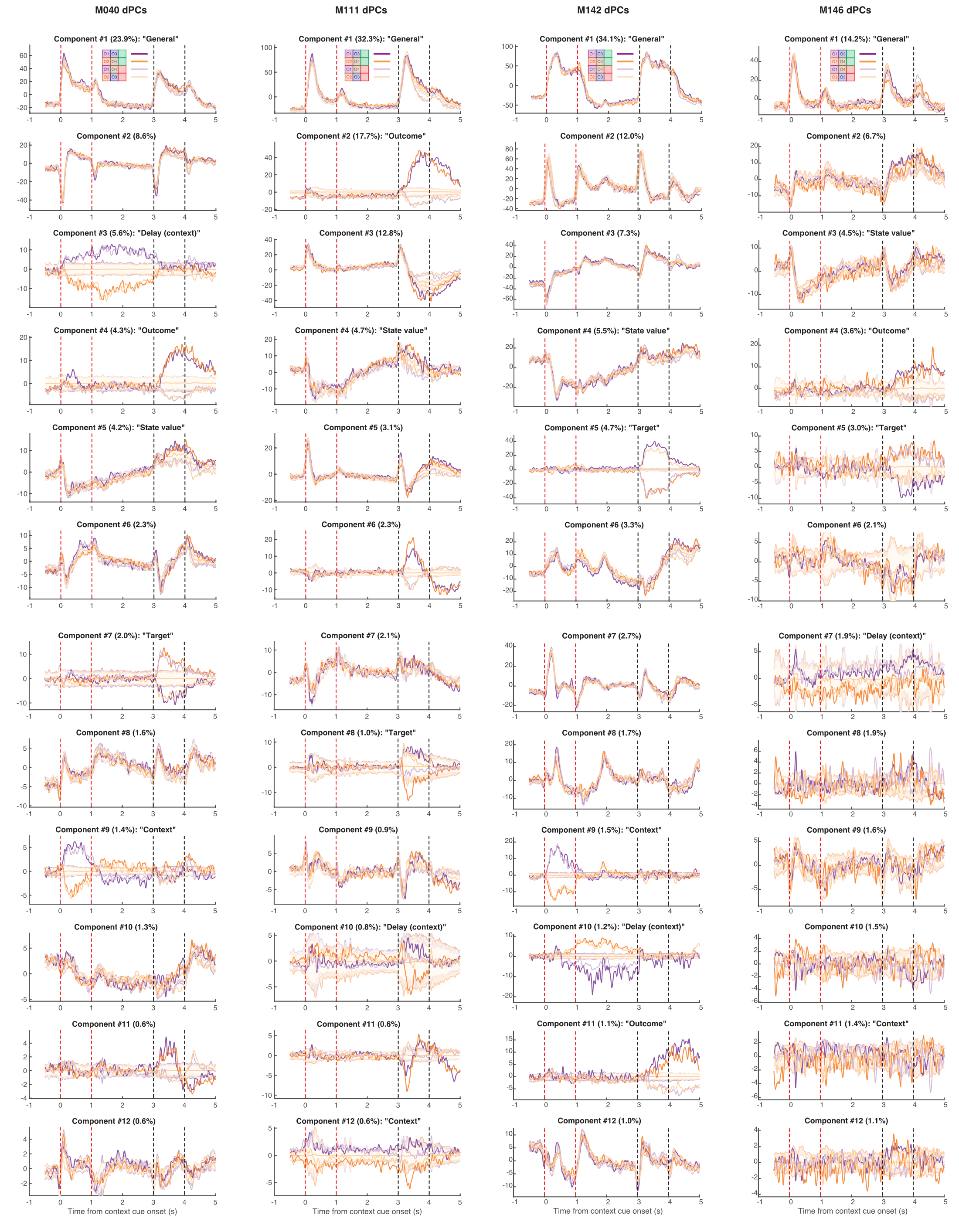

Supplement: S3 Fig — Each plot represents the trial-averaged projected activity onto the top 12 components (rows) for each mouse (columns) for each trial type. Plot title denotes the overall ranking of the component and the amount of variance explained by the component. Red lines border context cue presentation, and black lines border target cue presentation. From left to right shows components for M040, M111, M142, and M146. Components are ordered by amount of variance explained and include the following: a condition-invariant signal that responded to all odors (“general” cue component); a condition-invariant component present in most mice that showed a ramping-like activity after context cue onset, with a separation between rewarded and unrewarded trials after target cue onset (“state value” component); the context-related component that best separated context cues during the delay period (“delay (context)” component); the context-related component that best separated context cues during cue presentation (“context” component); the top target-related component that separated between target cues during target cue presentation (“target” component); and the top component that separated rewarded and unrewarded trials during target cue presentation (“outcome” component). Data: https://gin.g-node.org/jgmaz/BiconditionalOdor. (TIFF) [file pbio.3001338.s003.tiff]

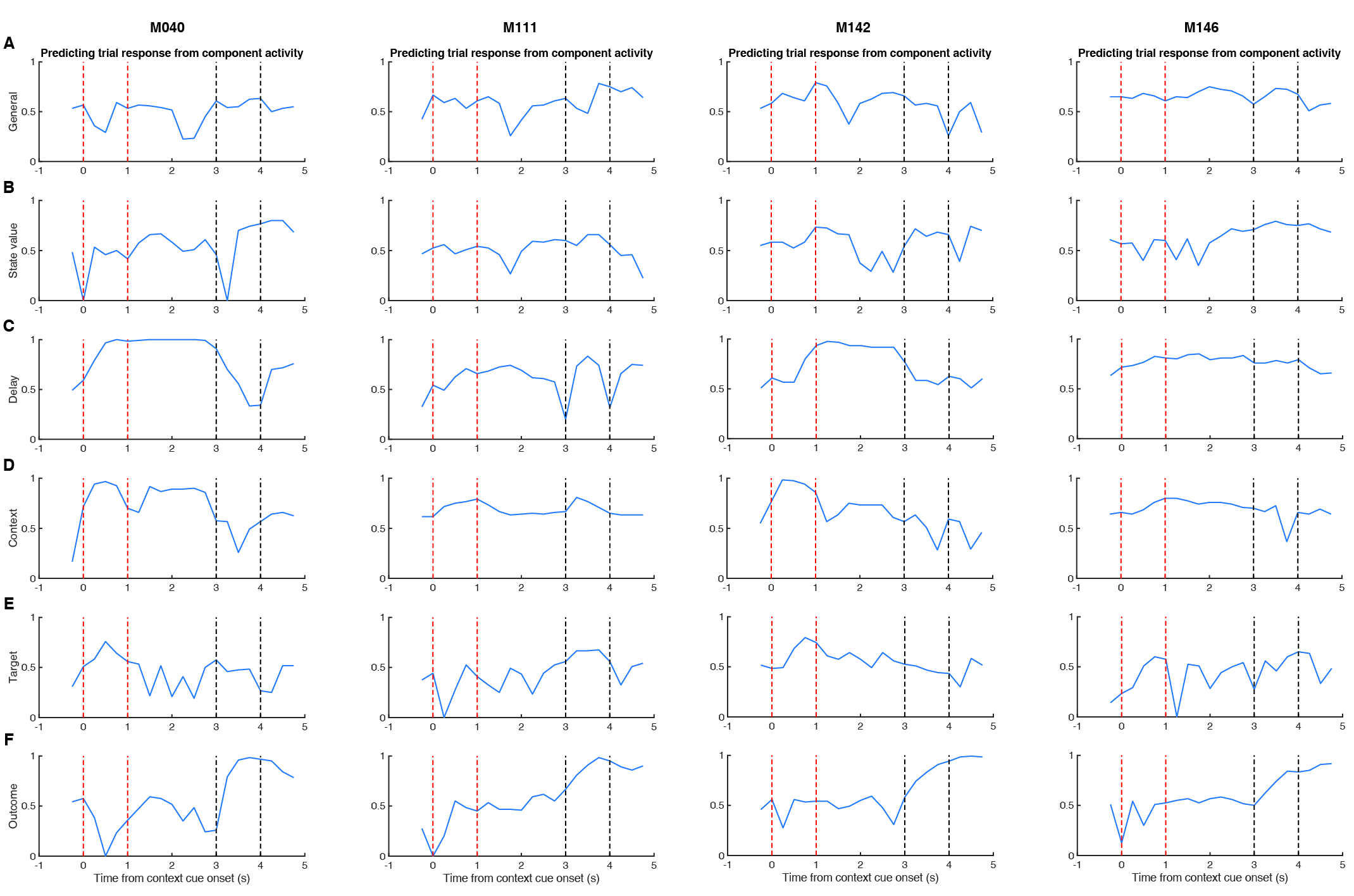

Supplement: S4 Fig — Each plot represents the accuracy of the behavioral prediction for a given component (rows) for each mouse (columns). Red lines border context cue presentation, and black lines border target cue presentation. From left to right shows predictions for M040, M111, M142, and M146. (A) Prediction accuracy for the general cue component. (B) Prediction accuracy for the state value component. (C) Prediction accuracy for the context-related delay component. (D) Prediction accuracy for the context component. (E) Prediction accuracy for the target component. (F) Prediction accuracy for the outcome-related target cue component. Data: https://gin.g-node.org/jgmaz/BiconditionalOdor. (TIF) [file pbio.3001338.s004.tif]

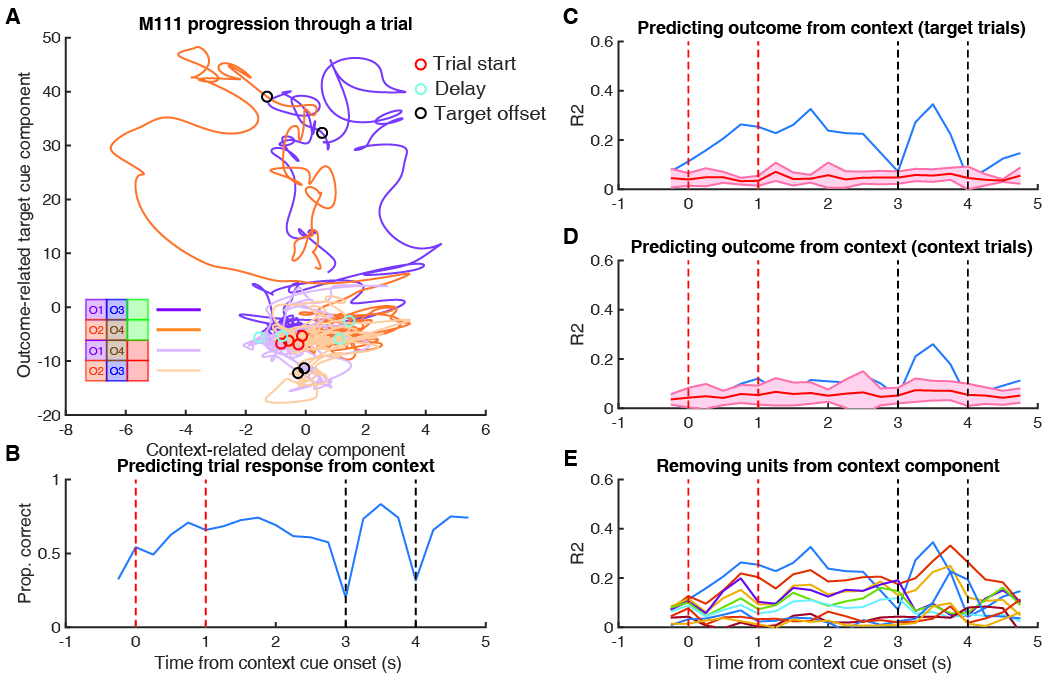

Supplement: S5 Fig — Shown is the relationship between the context-related delay component and the outcome-related target cue component for M111. (A) Progression of neural activity through a trial for each trial type in a two-dimensional neural subspace, with the trial-averaged projected activity in the context-related delay component (see Fig 7C) on the x-axis, and the trial-averaged projected activity in the outcome-related component (see S3 Fig) on the y-axis. Note the relatively weak structure in the context-related delay axis, compared to M040. Red circles signal context cue onset; cyan circles signal delay period 1 s after context cue offset; black circles signal 1 s after target cue onset. (B) Predicting behavioral response for a given target cue based on projected activity along the context-related delay component at various time points. Red lines border context cue presentation, and black lines border target cue presentation. (C) Predicting projected activity along the outcome-related axis after target cue onset for a given target cue (black circles from A) based on projected activity in the context-related axis at various time points. (D) Control analysis predicting projected activity along the outcome-related axis after target cue onset for a given context cue based on projected activity in the context-related axis. (E) Iteratively removing the top 10% of contributors to the context-related delay component and attempting to predict outcome-related activity as in C. Data: https://gin.g-node.org/jgmaz/BiconditionalOdor. (TIF) [file pbio.3001338.s005.tif]

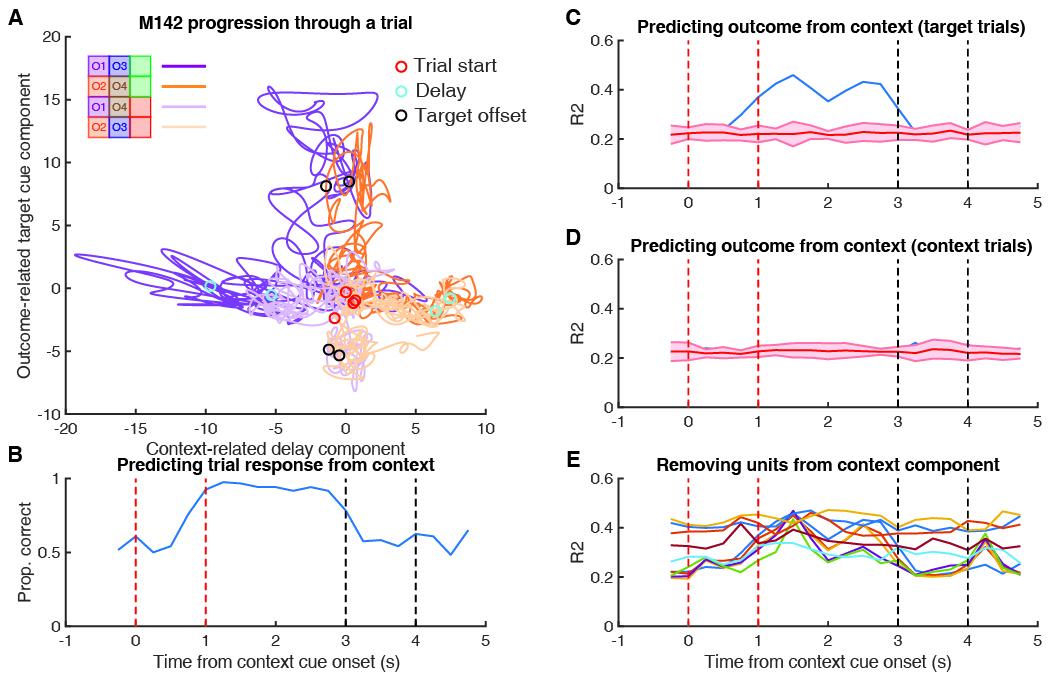

Supplement: S6 Fig — Shown is the relationship between the context-related delay component and the outcome-related target cue component for M142. (A) Progression of neural activity through a trial for each trial type in a two-dimensional neural subspace, with the trial-averaged projected activity in the context-related delay component (see Fig 7C) on the x-axis, and the trial-averaged projected activity in the outcome-related component (see S3 Fig) on the y-axis. Throughout the progression of a trial a separation is observed along the context axis, which then flows into the value axis after target cue presentation, similar to M040. Red circles signal context cue onset; cyan circles signal delay period 1 s after context cue offset; black circles signal 1 s after target cue onset. (B) Predicting behavioral response for a given target cue based on projected activity along the context-related delay component at various time points. Red lines border context cue presentation, and black lines border target cue presentation. (C) Predicting projected activity along the outcome-related axis after target cue onset for a given target cue (black circles from A) based on projected activity in the context-related axis at various time points. (D) Control analysis predicting projected activity along the outcome-related axis after target cue onset for a given context cue based on projected activity in the context-related axis. (E) Iteratively removing the top 10% of contributors to the context-related delay component and attempting to predict outcome-related activity as in C. Data: https://gin.g-node.org/jgmaz/BiconditionalOdor. (TIF) [file pbio.3001338.s006.tif]

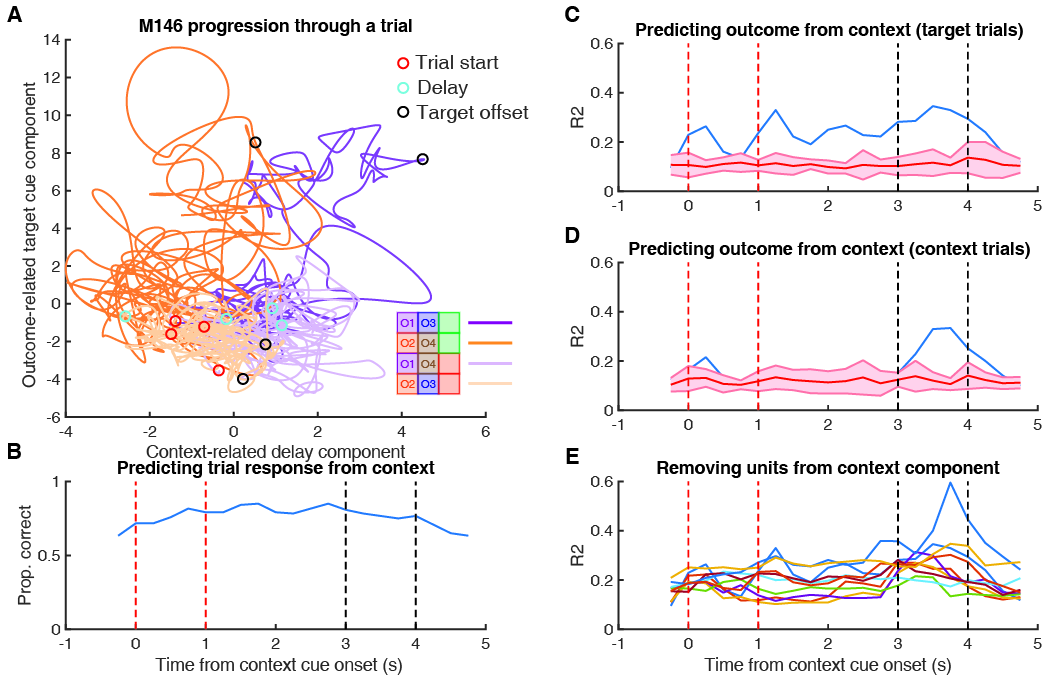

Supplement: S7 Fig — Shown is the relationship between the context-related delay component and the outcome-related target cue component for M146. (A) Progression of neural activity through a trial for each trial type in a two-dimensional neural subspace, with the trial-averaged projected activity in the context-related delay component (see Fig 7C) on the x-axis, and the trial-averaged projected activity in the outcome-related component (see S3 Fig) on the y-axis. Note the relatively weak structure in the context-related delay axis, compared to M040. Red circles signal context cue onset; cyan circles signal delay period 1 s after context cue offset; black circles signal 1 s after target cue onset. (B) Predicting behavioral response for a given target cue based on projected activity along the context-related delay component at various time points. Red lines border context cue presentation, and black lines border target cue presentation. (C) Predicting projected activity along the outcome-related axis after target cue onset for a given target cue (black circles from A) based on projected activity in the context-related axis at various time points. (D) Control analysis predicting projected activity along the outcome-related axis after target cue onset for a given context cue based on projected activity in the context-related axis. (E) Iteratively removing the top 10% of contributors to the context-related delay component and attempting to predict outcome-related activity as in C. Data: https://gin.g-node.org/jgmaz/BiconditionalOdor. (TIF) [file pbio.3001338.s007.tif]
