# Supplementary material for: Context coding in the mouse nucleus accumbens modulates motivationally relevant information
Source: PLoS Biol. 2022 Apr 29;20(4):e3001338. doi: 10.1371/journal.pbio.3001338 (PMC9094556; doi:10.1371/journal.pbio.3001338)
Supplement: Attachment — Submitted filename: MvdM_vStrContext_rebuttal.pdf [file pbio.3001338.s008.pdf]

Dear colleagues,

Thank you for your thoughtful comments on our manuscript, “Contextual gating of motivationally-relevant stimuli in the mouse nucleus accumbens”. A number of common threads emerged from your reviews, summarized here, along with an overview of the additions and changes we have made in response. A detailed point-by-point reply follows below.

**Review theme #1**: to what extent does this discrete-cue biconditional discrimination task really examine *context*?

- R1: “odor after-images” could persist throughout the delay, creating a compound sensory cue
- R2: are your context cues actually occasion-setters? What have the animals learned about these cues, as demonstrated by licking patterns?

We follow Bouton (2010) in using the term “context” to refer to any circumstance that changes the meaning of a target stimulus. Our task models this general idea using discrete cues, a trace of which is necessary in order to respond correctly to the target stimulus that follows. At the semantic level, we believe this application of the term “context” is in line with common usage, including recent papers using related tasks (see e.g. Fraser & Janak 2019, Wu et al. 2020) and captures the functionally relevant characteristics of (a) persistence, and (b) impact on the meaning of otherwise ambiguous cues. <u>We now include justification for our initial usage of the term “context” (in the Introduction, lines 61-62, and Results, lines 72-74) in this vein</u>.

However, your review comments also highlight that what we mean by “context” is not just a semantic issue: at the process and mechanistic levels, there are a number of different ways in which the context cues may affect the behavioral response to the target. These include occasion-setting, participation in configural cues, and direct association with the US. <u>To address this issue, we have included a new analysis of licking data (new Figure 1E and Figure 1 Supplement) and included a new Discussion section outlining these possibilities (Discussion, p. 21, “Context coding in the NAc”)</u>. In brief, we think that (1) the licking data does not support the possibility of direct association between the context cues and the US, and (2) although we cannot rule out the configural explanation, some properties of our experimental design and the neural data seem to favor the occasion-setting interpretation.

Addressing this issue in the expanded *Discussion*, we now explain that both the occasion-setting and configural accounts are plausible process-level implementations of the general notion of “context”, and that both may play a role in the behavioral and neural data we present. A dissection of their relative contributions must await future work, but we do not regard this as necessary for the demonstration that ~25% of NAc neurons encode cues that are of equal value but signal distinct task states required for correct responding to otherwise ambiguous cues.

**Review theme #2**: what do you mean by “value”, and does your task actually enable you to study it?

We use the term "value" as generally accepted in the reinforcement learning and sense of "magnitude of expected, discounted future reward" (we now define this explicitly at the start of the Results, line 80). The cues in our task signal distinct task states, some of which have equal value (e.g. the two context cues) and some of which do not (e.g. a given target cue being rewarded in one, but not the other, context). This general definition of value intentionally encompasses many potential sources of what the reward expectation may be based on, such as different task cues and their relationship, specific actions such as licking and their associated reward history, and so on.

The first major point of our study, the demonstration that NAc neurons encode context, relies critically on the two context cues (and associated task states) having equal value. We accomplish this firstly by design: each context cue is followed by exactly the same number of each target cue and availability of reward, and secondly by verifying that the actual number of rewards obtained does not differ between the two context cues (Results, lines 89-91). Thus, regardless of the specific sources of how the animals come to their value estimates (expectation of future reward) and what cues/actions they are associated with, these values are balanced between the two context cues and thus cannot explain the observed context signal.

In the second major point of the study, we show that this novel context signal predicts the subsequent neural and behavioral response to the target cue. In the original version of the manuscript, we interpreted the neural response as "value coding" in the sense that it discriminated between rewarded and non-rewarded trials (based on prior work demonstrating such coding in the NAc, e.g. Schultz et al. 1993; Roesch et al. 2009). However, as R2 points out, our experimental design cannot cleanly dissociate value coding from motor preparation. We leave it to future work to determine the specific nature of the signal(s) modulated by context coding, and conclude for now that the context signal predicts a behaviorally-relevant neural response. Thus, we have changed the terminology we use to "outcome-related" and "behaviorally-relevant" throughout the text, and expanded on the possible interpretations in the Discussion (p.23, "Relationship between context and behaviorally-relevant coding in the NAc").

**Further changes**:
- Increased robustness of dPCA analysis by adding new shuffles in response to R3 (revised Figure 7, and Figure 7 supplement 1)
- Minor edits throughout the text for clarity

Thank you for your consideration,

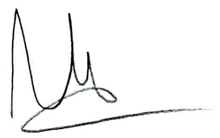

Matt van der Meer & Jimmie Gmaz

**Point-by-point reply** (verbatim reviews reproduced in blue with key points underlined, our responses in black):

**Reviewer #1**: In this study, Gmaz and van der Meer recorded activity in the NAcc of head fixed mice while they executed a two-step odor-guided task in which an initial set of contextual cues (O1 or O2) indicated if a subsequent target cue (O3 or O4) would be rewarded or not. Since the NAcc has been traditionally (albeit not exclusively) linked to value coding, neuronal responses to the contextual cues should be similar, as they are associated with the same potential reward. However, the authors found a subset of NAcc neurons that discriminated between the contextual cues, i.e. they seemed to encode for abstract elements of task structure rather than just how much reward the mice should expect. These results implicate the NAcc in more complex cognitive representations than would have been expected, especially in mice.
It was a pleasure to read this manuscript. The hypothesis is relevant to the field, the findings are interesting, and the presentation is crisp and clear. However, I see a couple of issues that need to be addressed to support the author's conclusions.

Thank you for the kind words, much appreciated.

Major comments:

- Studies with contextual cues typically employ cues of different sensory modalities (e.g. auditory/visual, visual/tactile, auditory/olfactory, etc.) to avoid potential compound cue representations. The authors only used odor cues, and partially addressed this potential confound. However, they argued as if this issue could arise only due to vestigial odor exposure during the delay. This is not the only concern, especially for odor cues. Transient exposure to odorants leads to persistent changes in firing in the olfactory bulb that can last for several breathing cycles (e.g. Patterson et al, PNAS 2013). It could very well be that the primary sensory representation of O3 and O4 is affected by persistent neural activity changes due to O1 or O2, and therefore the mice experience them as perceptually distinguishable cues. In effect, this would mean that the mice could be solving the task not by changing the value accrued to O3 and O4 based on the context, but rather by associating different percepts with the reward, i.e. O3'→ go, O3" → no go, O4'→ no go, O4"→ go. Persistent odor "after images" could explain the decoding of contextual cues during the delay period, and the fact that O3' and O3" would be distinguishable between themselves but still are more perceptually similar than O4' and O4" would explain the decoding of target odors from each other. Therefore, the arguments presented by the authors are insufficient to rule out this alternative explanation as unlikely.

We agree that it is possible that an after-image of the context cues forms a configure with the target cues, which would be sufficient to solve the task. Indeed, along with occasion-setting (i.e. changing the value of the target cues depending on context) this "configural" account is one of the two main process theories for context-dependent behavior. As you correctly point out, we did not do this account justice in the interpretation in our original manuscript. In this revision, we follow Bouton (2010) in starting from a general, "functional" definition of context, i.e. any circumstance that modifies the meaning of a specific target stimulus, which is agnostic to the specific associative process or neural mechanism underpinning it. In this view, the configural

and occasion-setting accounts are distinct, non-exclusive ways to satisfy this general functional requirement, and thus valid ways of conceiving of how context-dependent behavior may be accomplished. In the Discussion, we now write (p. 21):

*"We modeled context in our study by the simplest possible implementation: a discrete cue that signals distinct task states with separate stimulus-outcome associations for subsequently presented stimuli (Fraser & Janak, 2019; Wu et al. 2020). In general, the term ``context'' can refer to any circumstance that changes the meaning of a specific target stimulus (Bouton, 2010), as illustrated by the behavior of our subjects in emitting a different response (lick, no-lick) to a given target cue depending on the identity of the preceding context cue. However, this definition does not specify the particular process or mechanism through which the context cue comes to modify the response to the target. Two prominent, non-exclusive possibilities are (1) the context modifies the association between the CS and US (``occasion-setting'') and (2) the context forms a configural cue with the CS, which then gets associated with the US. In addition, (3) the context cues may enter into direct association with the US themselves (Trask et al. 2017; Delamater et al. 2017). Because we observed no licking CR in response to the context cues, (3) seems unlikely to be a major contribution. Although our experimental design incorporated a delay between the presentation of the context and target cues, impairing the ability of the context cue to enter into a configure with the target, we cannot entirely rule out the possibility that some neural trace of the context cue remains after its offset (see the discussion in the next paragraph). Thus, our experiment does not allow us to determine the precise contributions of (1) and (2), which could be addressed in future work with behavioral experiments such as extinction and transfer tests, and perhaps representational similarity analyses on the neural data that compare across elemental and configural cues. Importantly, our conclusions in this study do not depend on (1) or (2) being the case; either way, our data demonstrate the existence of a context signal in the functionally important sense that it is value-independent and predictive of the subsequent response to a given target cue."*

Note that in this revision, we also included analysis of licking data during the context cues to address a possibility raised by Reviewer 2 that the context cues themselves enter into associations with reward (discussed in more detail in the response to R2 below).

- Following the previous issue, given that there was just one pair of contextual cues and the analyses were all within subjects, and that it is possible than the mice are solving the task without necessarily using the "contextual cue" to actually gate context coding, then <u>it is still also possible that the discrimination of O1 and O2 may reflect the sensory identity of the different cues</u>. I agree with the authors that even if this is the case, it still means that NAcc neurons encode information that goes beyond value, which is the most poignant point of the study. But the authors should provide stronger arguments or data as to why their particular interpretation is more likely.

We agree: from our particular experimental design, which modeled context using the simplest possible implementation, we cannot determine with certainty to what extent the context signal reflects the sensory identity of the context cues, and/or signals an abstract task state. As with

our response above, we take the view that either is a valid implementation of the functional idea of a context -- in the sense that the signal is (a) persistent after cue offset, and (b) predictive of the subsequent response. We also note that because for some units, context sensitivity emerges only during the delay (e.g. the example in Figure 2F), simple persistence of the original sensory response cannot be a full account of the data. Thus, we now write in the Discussion (p. 21-22):

*"What is the nature of the context signal in the NAc? Our results show that the activity of a significant proportion of single units (26% across mice) discriminates between the two context cues, but this finding does not by itself specify the information encoded by this signal. In particular, since we only used one cue per context, the identity of that cue itself could serve as the context signal. While we believe that the 2 s of active flushing of the odorant during the delay between cues is sufficient for the context cue odor to disperse from the experimental apparatus, previous work suggests that the olfactory bulb maintains an ``after-image" of the odorant (Patterson et al. 2013), and thus we cannot exclude the possibility that a representation of the context odor remained and combined with the target cue to form a configural cue. However, we think this is unlikely to be a complete account of the data, given the presence of single-unit and population-level correlates that only discriminated between context cues during the delay period following context cue offset. A different possibility is that the context signal encodes an abstract task state independent of the sensory properties of the context cue(s); this idea could be probed by having multiple distinct cues signal the same context and testing whether cue identity or context identity is a better match for the neural response. However, in either case, the signal retains the functionally important properties of a context in modifying the subsequent response to a given target cue."*

Minor comments:

- It is very inconvenient to have to rotate the page to read figure 6. It would be best to rescale, or perhaps simplify, so it would fit in the standard portrait view.

Thank you for this suggestion, we reformatted this figure so it could be rotated in the standard portrait view.

- Is there any particular reason why only females were used? Are they easier to head fix? That is true for rats, but I wonder if it's the case for mice as well.

We trained equal quantities of male and female mice, but only the female mice learned the task to criterion in order to proceed to recording. We now explain this in lines 449-450 in the *Methods*.

**Reviewer #2**: The manuscript by Gmaz et al. investigates how a non-value-based signal can predict the value of a subsequent cue-reward association through neuronal activity signatures in the nucleus accumbens. The authors designed a behavioral task in which mice receive a neutral cue that informs the rules of the task when subsequent cues are presented. Importantly, there is a delay period between the context cue and the onset of the cue, thus requiring a representation

of this information to be prolonged. When they record single unit responses in the NAc to different aspects of this task, including what they term contextual cue coding, coding during the delay period, target cue coding, and value coding after target cue onset. The authors conclude that the NAc responds to various features of the task and can retain information about the neutral cue in the delay window before target cue onset. In addition, the authors looked at population-level activity and utilized dPCa to extract latent factors relating to the context cues, target cues, and trial value. They identified that the context signal extracted via dPCa is in fact predictive of the value encoded in the association between the target cue and reward.

Overall, the data are interesting and informative and my enthusiasm for the data and results is high; however, there are several issues - some semantic and some experimental - that should be addressed before publication. The biggest issue I have with the manuscript is how the word value and non-value are used. The argument is that the cue that the authors term contextual does not have value; however, there is not data to show whether those cues do or do not have value. Further, while the authors call these cues contextual, I would argue that those cues serve as occasion setters - rather than contextual cues.

Thank you for the encouraging words and the insightful comments; please see below for specific responses.

Further, the task is entirely reward-based, so it is unclear if the responses to the reward itself are valence or other aspects of learning and behavior such as salience, which would not be able to be parsed in a purely reward-based task.

Overall, the data are interesting, but the manuscript would benefit from more data showing that the contextual cue does not in fact acquire conditioned valence and more precision are care with speaking about the specific findings based on the data at hand.
I have listed my specific comments below as major and minor.
Major
1. The biggest weakness of the entire manuscript is the lack of behavioral data analysis showing whether the cue that the authors term contextual is actually a contextual cue (which would acquire value from the task) or an occasion setter (which by definition does not acquire value on its own). Regardless of whether the cue has intrinsic positive value because it signals that subsequently reward will be delivered, intrinsic negative value because it is a conditioned inhibitor of the incorrect response, or whether it is an occasion setter is irrelevant to the overall data, but is critical to the conclusions the authors are trying to make. Therefore, they should add behavioral data showing that 1. The animals learn this cue specifically and 2. Whether the cue does or does not have value itself.

As we explain in the response to Reviewer 1 above, we take the view that the term “context” has an admittedly vague, but generally accepted, meaning of “any circumstance that modifies the meaning of a specific stimulus” (Bouton, 2010), which emcompasses multiple, more specific processes and mechanisms such as occasion-setting and configural cue formation. To keep the manuscript accessible for a more general audience, we elected to keep the general term

“context” as a functional definition, but we now take care to explain that (1) we model this general concept with a specific task design (line 61, line 72), and (2) the general notion of “context” may refer to multiple processes, which we review and relate to our experiment in the Discussion (reproduced in response to your next point, directly below).

Here are some experiments that should be included - many of which only require reanalysis of the original behavioral data:

o a raster plot of responses to the various trials would be appropriate to determine if mice are just making a couple of errant licks on the trials in which no reward is given or if there are actual lick bursts.

Thank you for this excellent suggestion. We have added analysis of the licking data (Figure 1E, p. 8; Figure 1- supplement 1, p. 34):

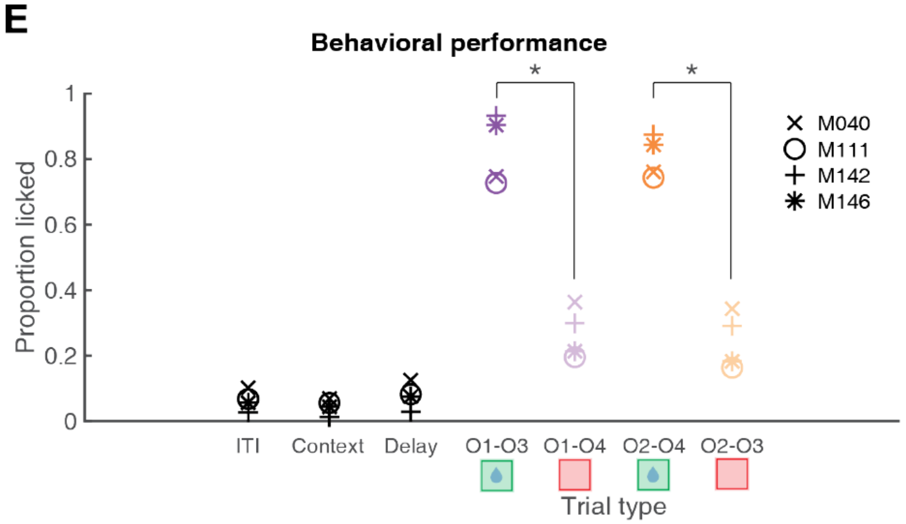


**Figure 1E**: Behavioral performance during recording sessions showing the average proportion of trials with a licking response during the inter-trial interval (ITI), context cue period, delay period, and target cue period for each trial type for each mouse, demonstrating that mice discriminated between rewarded (green) and unrewarded trial types (red). Asterisks denote significant differences ($p < 0.05$ based on a bootstrap with trial labels shuffled, see *Methods*).

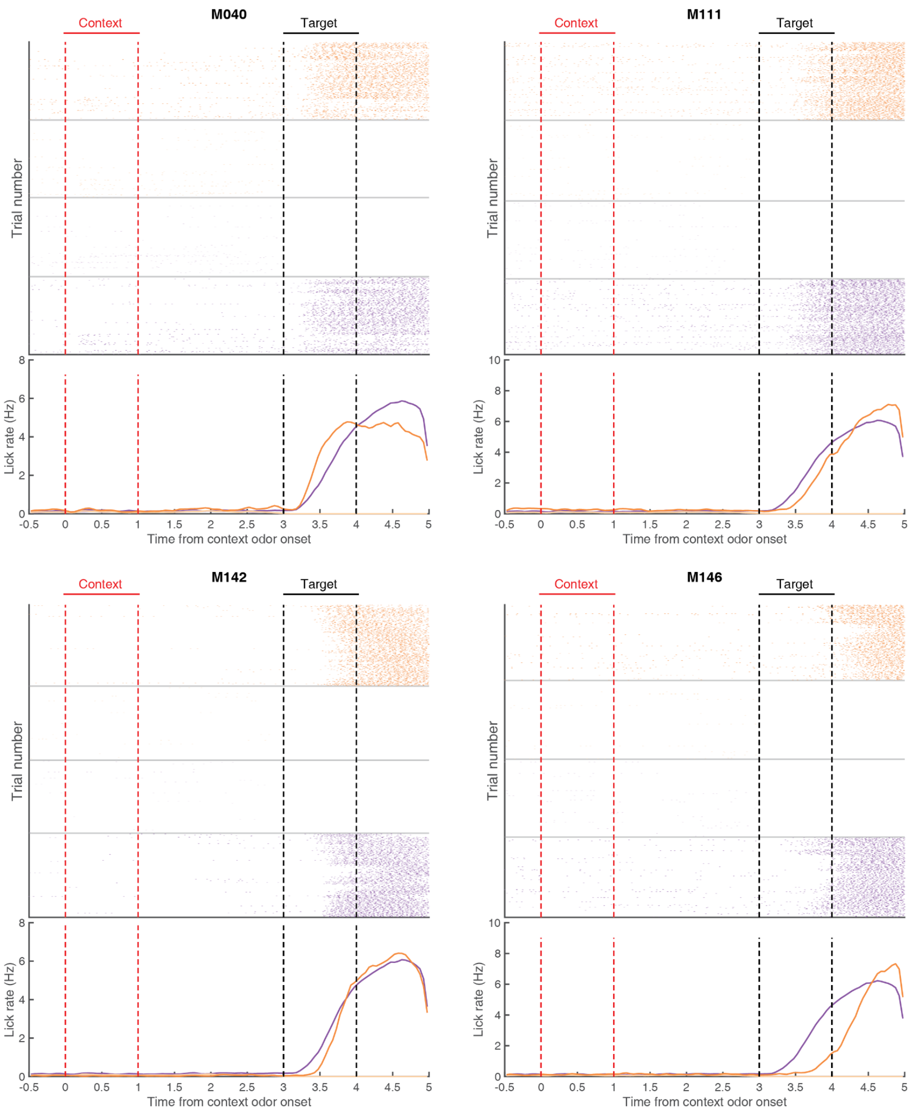


**Figure 1 supplement 1**: Average rate of licking during correct trials for each mouse across all recording sessions. Top of each plot shows lick rasters during correct trials for the four trial types for all recording sessions (purple: trials with context cue O1; orange: trials with context cue O2; dark colors: rewarded trials; light colors: unrewarded trials). Bottom half of each plot shows trial-averaged licking rates for each trial type aligned to context cue onset. Data shown is averaged across all recording sessions. Context cue presentation (0-1 s) is indicated by red lines, and target cue presentation (3-4 s) is indicated by black lines. Note that for correct trials, substantial licking only appears after target cue onset, and for rewarded trials only.

The above figures and associated statistical analysis (described in the Results, lines 93-95) demonstrate that there is no increase in licking in response to the context cues compared to the intertrial interval. Thus, based on the absence of a CR, there is no evidence that the context cues themselves have entered into a direct association with the US. We now point this out in the Discussion, which also includes reference to other possible implementations of context at the process and mechanistic levels (p. 21):

*“In general, the term ``context'' can refer to any circumstance that changes the meaning of a specific target stimulus (Bouton, 2010), as illustrated by the behavior of our subjects in emitting a different response (lick, no-lick) to a given target cue depending on the identity of the preceding context cue. However, this definition does not specify the particular process or mechanism through which the context cue comes to modify the response to the target. Two prominent, non-exclusive possibilities are (1) the context modifies the association between the CS and US (``occasion-setting'') and (2) the context forms a configural cue with the CS, which then gets associated with the US. In addition, <u>(3) the context cues may enter into direct association with the US themselves (Trask et al. 2017; Delamater et al. 2017). Because we observed no licking CR in response to the context cues, (3) seems unlikely to be a major contribution.</u> Although our experimental design incorporated a delay between the presentation of the context and target cues, impairing the ability of the context cue to enter into a configure with the target, we cannot entirely rule out the possibility that some neural trace of the context cue remains after its offset (see the discussion in the next paragraph). Thus, our experiment does not allow us to determine the precise contributions of (1) and (2), which could be addressed in future work with behavioral experiments such as extinction and transfer tests, and perhaps representational similarity analyses on the neural data that compare across elemental and configural cues. Importantly, our conclusions in this study do not depend on (1) or (2) being the case; either way, our data demonstrate the existence of a context signal in the functionally important sense that it is value-independent and predictive of the subsequent response to a given target cue.”*

- Do the mice primarily respond in the phase immediately following the target cue onset or after the 1 second presentation? I would also like to see inter-trial interval licks. Do the mice show any indiscriminate licking? Do they lick at all in the delay period?

We have added the licking plots as suggested above (Figure 1E and Figure 1 - Supplement 1), showing that licking remains low during the ITI, context cue presentation, and the delay, starting only during presentation of the target cues.

- Show data to show that the mice to not lick in response to the first cue that is presented - i.e. there is no anticipatory licking and this cue has not acquired value.

The above plots demonstrate that there is no anticipatory licking, either in response to the context cues, or during the subsequent delay period.

- It would also be important to show that the first cue is not a conditioned inhibitor of the secondary negative response. To do this you could present this cue concurrent with another CS+ and show that it does not reduce the rate of responding.

We take the reviewer’s point to be that one way the context cues could come to control the response to a rewarded target cue is by conditioned inhibition of a “not-licking” response. While we agree this is a theoretical possibility, we think it is unlikely, primarily because of the way in

which performance develops over the course of training. Mice initially tend to lick in response to all target cues, and gradually learn to withhold responding on unrewarded trials. This evolution follows that which has been generally observed in similar tasks such as negative occasion-setting and negative patterning (e.g. Holland, 1992) and does not seem consistent with conditioned inhibition of a withholding response. As with the other process-level explanations we discuss above (and in the revised Discussion), the extent to which these processes play a role does not change our overall conclusion that functionally speaking, there is contextual modulation of the response to a given target stimulus.

2. In the paragraph following line 123, that authors state that the number context-coding units related to the number of training sessions. If you continued training sessions in mouse M111 until it reached at least 20 sessions, the authors would address whether the variability in context coding is in fact related to the number of training sessions rather than precise recording location or a factor of only needing so many context-coding units to maintain representation of the context during the delay.

Thank you for the suggestion; we agree that this is an interesting issue, one that would ideally be tested with a dedicated experimental design that could track how the strength of context coding develops over the course of training, and ideally also with interventions that could test the contribution of NAcc at these different stages (we are currently piloting an optogenetics experiment testing this). In an attempt to make the most of the data we already have, we tested whether the proportion of context-coding units differed between the first and second half of recorded neurons for each mouse. We found some differences on an individual animal basis, but the direction of the change was not consistent across animals (percentage of context-coding units found in each half of recording sessions for each mouse: M040: 14% for first half and 40% for second half; M111: 0% for first half and 10% for second half; M142: 44% for first half and 25% for second half; M146: 19% for first half and 23% for second half). Thus, based on this small sample, it is not obviously the case that more training equates to stronger context coding, so we leave this possibility open for future work to explore.

3. The manuscript suffers from an overall lack of precision in wording when talking about the data. For example, does value mean valence? The authors should go through and operationally define terms and use them appropriately. Along a similar line, I do not believe that the cues that the authors term contextual cues are actually contextual cues. Also the responses that the authors attribute to valence - i.e. reward responses - also may not be valence. In that situation many things are changing (reward prediction is met, salience is increased, valence goes up) and the experiments are not designed to parse these scenarios. The authors should take care to go through the manuscript to clearly state what they are talking about and how it fits into the previous work in the psychology field.

We see three important points here, which we will address in turn:

1) *does value mean valence?* We use the term "value" in what we believe is a commonly used sense of "expected, discounted future reward", reflecting the subjective utility assigned by the

subject to the various task states, actions and outcomes. We purposely use this very general definition, because for the purposes of this study, the main point is that the two context cues do not differ in the strength of their association with reward -- regardless of the precise underlying construct or process. We now define what we mean by "value" on its first use (line 80: "Thus, by design, rewarded trial types "O1-O3" and "O2-O4" both had the same outcome value (future expected reward), while unrewarded trial types "O2-O3" and "O1-O4" also had the same outcome value") and changed the wording throughout the manuscript for those situations where we cannot distinguish between value and other alternatives such as motor preparation (see our response to your point 3, below).

2) *are the cues we call "context cues" actually contextual cues?* We now write when introducing this term in the Introduction (lines 61-64): "...we model context as a task state signaled by one of two discrete odor cues. Specifically, animals were presented with two different "context" cues that determined whether a subsequent "target" cue would be rewarded (Figure 1A)." A few lines down, in the Results, we justify this choice of terminology further, and point to a more extended discussion: "We use the term "context" here to mean a cue that modifies the meaning of a subsequently presented target cue (i.e. whether that target cue predicts reward or not; see *Discussion*)" (lines 72-74). The corresponding section of the Discussion is quoted above (on page 9 of this rebuttal). As argued there, we acknowledge that the term "context" is sometimes used in a specific, technical sense, and outline some of those in the Discussion; however, I hope you will agree that "context" also has a more general meaning, used as a starting point in the influential Bouton (2010) review, which is helpful in communicating the intuition behind our task and associated analyses to a wider readership.

3) *the responses that the authors attribute to valence - i.e. reward responses - also may not be valence*. We agree. In the second major point of the study, we show that context coding predicts the subsequent neural and behavioral response to the target cue. In the original version of the manuscript, we interpreted the neural response as "value coding" in the sense that it discriminated between rewarded and non-rewarded trials. This interpretation was based on prior work demonstrating such coding in the NAc (e.g. Schultz et al. 1993; Roesch et al. 2009). However, as you correctly point out, our experimental design cannot cleanly dissociate value coding from motor preparation. We leave it to future work to determine the specific nature of the signal(s) modulated by context coding, concluding for now that the context signal predicts a behaviorally-relevant neural response. Thus, we have changed the terminology we use to "outcome-related" and "behaviorally-relevant" throughout the text, and expanded on the possible interpretations in the Discussion (p.23, "Relationship between context and behaviorally-relevant coding in the NAc").

Minor

4. Line 97, "that" repeated twice

Fixed, thank you.

**Reviewer #3**: In this interesting study, Gmaz and Van der Meer ask whether nucleus accumbens (NAc) neurons are responsive to context cues that establish the values predicted by subsequent "target" cues, and whether NAc neurons code for the value indicated by the combination of a prior context and current target cue. They used a simple behavior task in which the appropriate response to a target cue (go or no go) depended on the prior context cue; a delay was interposed between context cue and target cue presentation. They record from a large population of neurons in four mice, and a very strong feature of the study is that all results are reported using data from individual mice, with differences in behavior across mice described and related by the authors to differences in neural coding. In the simplest analyses, the authors find that the context cue delay period firing of a subpopulation of 5 to 13% of neurons significantly predicted the behavioral response to the target cue, and that pseudopopulation activity during this period also predicted the behavioral response above chance. The authors then apply more sophisticated population coding analyses to test whether information coding consistent with three hypothesized functions could be detected in the pseudopopulations. These functions are context coding (firing is different for different context cues until target cue presentation), state value encoding (coding of temporally-discounted reward value, which should ramp upwards from context cue presentation regardless of cue and then differ markedly as soon as a go (rewarded) vs no go (non-rewarded) target cue is presented), and what the authors call a "general cue response", which is essentially a firing response to any cue without differentiation according to meaning. They find that the "general cue response" is most strongly coded, but they also find evidence for context and state value coding. Finally, they find that context coding (during the context cue/delay period) predicts subsequent value coding (during/after target cue presentation), consistent with a gating mechanism in which the context activity modifies the subsequent target activity so that it appropriately predicts value (and/or selects the appropriate behavioral response).

This study was carefully executed and thoughtfully analyzed, and it addresses an important issue. The findings of context cue coding by NAc neurons are novel and exciting. There are however a few issues that should be resolved.

Thank you for the clear summary and the encouraging comments. We would also like to note the most straightforward finding of the study, which is that 26% of neurons discriminated between the two context cues, in a way that cannot be explained by differences in expected value.

1. The major stumbling block I have to fully accepting the dPCA analysis is that the authors found many other components than the three they focus on (corresponding to general cue, state value and context coding), and these other components are only cursorily described. Two of the chosen components (state value and context) account for roughly 5% and 1-2% of the variance, and presumably the other components that are not shown each account for a similar degree of variance - or perhaps more. The impression is that the authors chose the components that happened to fit their hypothesis while ignoring the others. Perhaps some form of bootstrapping analysis would convince the reader that the components they identify with real data do not explain a similar degree of variance in randomized data. In addition, the authors should show

ALL of their components in a figure similar to fig 7-supplement 1, but sorted in order from first to last, with the degree of variance explained marked, and with the author's components of interest (general, state value, context) highlighted. This would allow readers to draw their own conclusions about the relative strength of coding for the components that match the authors' hypothesis.

Thank you for these important suggestions. We have added shuffles to the dPCA analyses, where we randomly permute the trial labels for each neuron (breaking any systematic relationship between firing rate and the identity of any of the cues, as well as any particular outcome) before generating the trial-averaged responses. We have added the output of this shuffle analysis onto the dPCA figures (Figure 7; Figure 7 - Supplement 1) to show that the dPCA components in the data explain more variance than expected by chance. Next, we have reconfigured Figure 7 - Supplement 1 to show the top 12 components for each mouse in order of descending amounts of variance explained, with components of interest highlighted, as suggested. We have also modified the Results to ensure we present the "big picture" first before discussing specific components of a priori interest (starting at line 192):

*"This analysis revealed a variety of time-varying components, capturing different aspects of the task (see Figure 7-supplement 1 for the top 10 components from each animal). We then compared the components extracted from the data to components from a shuffled distribution extracted by shuffling the trial identity of each trial (note, this process will preserve any general time-varying component that is independent of trial type). The strongest component across all animals was a non-specific time-varying signal whose activity and time course was related to the time course of the odor cues, and for this reason we call it the "general cue" signal…"*

2. There was no imposed delay between target cue presentation and the animal's "go" response. As such, any neural response after target cue presentation could be related to movement (or witholding movement) rather than to value. The authors seem to acknowledge this, but nevertheless use "value" terminology (particularly in their description of the post-target components as "value" components). In the Discussion they should mention the absence of a delay explicitly and describe how an experiment including a delay would allow value coding to be dissociated from movement coding.

We agree. In the original version of the manuscript, we interpreted the neural response as "value coding" in the sense that it discriminated between rewarded and non-rewarded trials. This interpretation was based on prior work demonstrating such coding in the NAc (e.g. Schultz et al. 1993; Roesch et al. 2009). However, as R2 also points out, our experimental design cannot cleanly dissociate value coding from motor preparation. We leave it to future work to determine the specific nature of the signal(s) modulated by context coding, concluding for now that the context signal predicts a behaviorally-relevant neural response. Thus, we have changed the terminology we use to "outcome-related" and "behaviorally-relevant" throughout the text, and expanded on the possible interpretations in the Discussion (p.23, "Relationship between context and behaviorally-relevant coding in the NAc"), including the idea that an experiment utilizing an explicit response delay period would offer a way to distinguish these accounts (lines 376-377)

3. The authors say their findings support the hypothesized "switchboard" function of the NAc (line 354). The simplest way this function could work is if a structure such as the prefrontal cortex elevated the activity of a subpopulation of NAc neurons in one context (and perhaps depressed it in other contexts), allowing certain cues to further activate the excited neurons to produce behavior while other cues are rendered incapable of exciting the population in that context. The authors claim that their "context" coding as in the form illustrated in fig. 4A is more or less consistent with this idea, but one would expect a different response to the two target cues depending on the context - something that is not apparent either in fig. 4A and appears to be quite messy in the "real" data in fig. 7c. This raises a broader issue: if context coding subsequently influences target cue coding by some mechanism that is not a simple change in firing rate or excitation, then what is that mechanism? Given how complex such a mechanism is likely to be, how much credence should we give the idea that even though there is a correlation between context and value encoding, the two are causally related? The Discussion is not clear on this point and could be expanded to at least speculate on mechanism -- and to emphasize that without any plausible mechanism hypothesized (much less established) for this function, the observation of a mere correlation should be interpreted with caution.

We agree with the reviewer that in the "switchboard" model we would expect a difference in how the NAc responds to the target cue based upon the context, and this is indeed what we find. Figure 7C referenced by the reviewer may have been misleading in that respect, because this panel shows the context-related component *during the context cue and delay period only* (most clearly visible in subjects M040 and M142). Because it averages over both target cues, it cannot be used to determine if the response to a given target cue depends on context. Instead, the analysis described in the section "Context-specific ensemble states predict the magnitude of the subsequent outcome-related response" (Results, p. 18) and associated plots (Figures 8C-E) are specifically meant to test this idea. We write (lines 257-263):

*"To test this, we trained a linear regression to predict the activity of the top outcome-related component during target cue presentation, from the delay context-related component, and found that the delay component could account for a significant amount of variability for the outcome-related component for a given target cue during the delay period (Figure 8C for M040 & supplements for M111, M142, M146; Proportion of variance explained at end of delay period, M040: 0.45; z-score: 63.16; $p < 0.001$; M111: 0.15; z-score: 7.25; $p < 0.001$; M142: 0.42; z-score: 10.92; $p < 0.001$; M146: 0.22; z-score: 8.89; $p < 0.001$)."*

Thus, we do find support for the hypothesis (illustrated schematically in Figure 4A) that the context cues move NAc ensemble activity into distinct neural states which can be used to predict the response to an otherwise identical target stimulus. Nevertheless, the reviewer's point that this relationship does not necessarily imply causation is well taken, and we have added notes of caution in the relevant section of the Discussion, reproduced below (p. 25):

*"A potential function for the context coding observed in the present study is implementation of the hypothetical "switchboard" function of the NAc (Gruber et al., 2009; Murer & O'Donnell,*

*2016), serving as a routing mechanism to enable dynamic value representations of target cues (gating; Figure 6B). Indeed, the distinct occupancy in the pseudo-ensemble space for each context cue signals that the context cues might be driving the NAc into separate network states, setting an initial state for subsequent input-output flow of the target cue. In addition to the presence of a context signal, this routing function would also require that the context signal is both functionally linked to the subsequent outcome signal, while simultaneously not interfering with outcome-related output. We found support for the former from the observation that activity in this space during the delay period could explain a significant proportion of variance in the behaviorally-relevant component during the target cue period. Context-specific cortical input that modulates the excitability of individual NAc neurons, resulting in a differential response to the subsequent target cue is a candidate mechanism for these population-level observations. Similar population-level observations have been observed in the field of motor control and, more recently, economic choice (Elsayed et al., 2016; Kaufman et al., 2014; Yoo & Hayden, 2019).*

*Furthermore, given that the context-related and behaviorally-relevant components were orthogonal from one another, it suggests that context coding does not interfere with the ability of downstream structures to read out outcome-related information. <u>However, given that our experiments were correlative in nature, these interpretations are speculative, and future work is needed to test the causal contributions of these components</u>. Finally, another potential functional role for the observed context coding is in forming the associations between reward-predictive cues and the rewards themselves. Recently, several studies have implicated cortico-striatal circuits in credit assignment (Oemisch et al., 2019; Parker et al., 2019), raising the possibility that this context signal may be used for learning to assign credit to the appropriate state. <u>Whether these context components are generated in the NAc or inherited from inputs, as well as if they represent an internal computation that is locally used to organize NAc activity, or are conveyed downstream, remains to be determined</u>."*

4. Fig. 3G shows a high number of "pre-context" coding units, but these units are not described in the Results and the observation is not interpreted. What exactly do these neurons significantly code for?

Thank you for bringing this to our attention. We have modified Figure 3G to clarify that these are units that discriminate between the pre- and post-cue period, in alignment with the text.

5. Line 167: "We found that 5-13% of units across time points were able to predict subsequent response to a target cue above chance" - did these units overlap to a large degree with the "context" coding units from Fig. 3? And what explains any degree of non-overlap?

Of the 80 context-coding units observed in the study, 52 (65%) contained predictive information about the animal's subsequent behavioral response. Likewise, of the 49 delay-coding units, 38 (78%) were predictive about the subsequent behavioral response. Given that context-coding is most useful for the animal during presentation of the target cue when it must make the appropriate behavioral response, it makes sense that there is a larger proportion of delay- over

context-coding units containing predictive information. We have added these numbers in the results section. Thus, as the Reviewer anticipated, there is a large degree of overlap between context-coding and subsequent behavioural prediction; any non-overlap is likely due to a combination of (1) the possibility that units do not distinguish between contexts but are nonetheless informative about the subsequent response, perhaps because they encode a quantity related to task engagement or motivation; and (2) differences in neural activity between different sets of trials used to distinguish between different contexts and different outcomes.

6. It would be useful if the figures showing time on the X axis had labels for the different events (context cue on/off, etc). The convention of red and black dashed lines eventually becomes clear but additional labels on the figures themselves would be much easier on the reader.

Thank you for this suggestion. We have added “context” and “target” labels to the main figures to help clarify trial structure as suggested.

7. Line 310 "yoked" has a specific meaning in animal behavioral science, which the authors don't intend here.

Thank you for pointing this out; we agree, and have changed “yoked” to “correlated”.

8. Typos in lines 72, 97. Line 317 fewer days, not less.

Fixed, thank you.

References:

Bouton, M. E. (2010) *The multiple forms of "context" in associative learning theory*. In B. Mesquita, L. F. Barrett, & E. R. Smith (Eds.), The mind in context (pp. 233–258). Guilford Press.

Holland, P. C., (1992) *Occasion setting in Pavlovian conditioning*. In Psychology of learning and motivation (Vol. 28, pp. 69-125). Academic press.
