# Supplementary material for: Context coding in the mouse nucleus accumbens modulates motivationally relevant information
Source: PLoS Biol. 2022 Apr 29;20(4):e3001338. doi: 10.1371/journal.pbio.3001338 (PMC9094556; doi:10.1371/journal.pbio.3001338)
Supplement: Attachment — Submitted filename: MvdM_vStrContext_rebuttal2.pdf [file pbio.3001338.s009.pdf]

Reviewer comments: (reproduced verbatim in blue)

Reviewer #3: The authors have addressed most of my concerns. However, the new bootstrapping analysis used in figures 7 and S3 leaves me confused. It's very hard to find the shaded areas of the plots that indicate the range of the results using shuffled data. Presumably that's because the range was quite narrow in some cases and overlapped with the results from unshuffled data. But if that's the case, the bootstrapping analysis shows that the results with unshuffled data don't differ much from the results from shuffled data despite the authors' conclusions to the contrary. I suspect that I am simply missing something and that a more thorough explanation of the bootstrapping results (or perhaps a better visual representation of the results from shuffled vs unshuffled analyses) would clarify it.

Thank you for raising this important point, which alerted us that we did not clearly articulate the intuition behind the bootstrap analysis, or explain how to interpret the associated results. The neural data recorded during our task contains a number of different components, including some that are specific to the particular cue used (e.g. those encoding the difference between context odor 1 vs. context odor 2; or the difference between rewarded target odor 3 vs non-rewarded odor 4), as well as other components that do not distinguish between the cues (e.g. a general “cue presence” response). Our bootstrap analysis, which shuffles trial labels (context 1 vs context 2) is designed to reveal the context-dependent component: any systematic difference that exists in the data between the two contexts will be destroyed by this shuffle. In contrast, cue-independent components such as a ramping signal up to target cue presentation, or a general cue presence signal, would be preserved by this shuffle. This is indeed what we see:

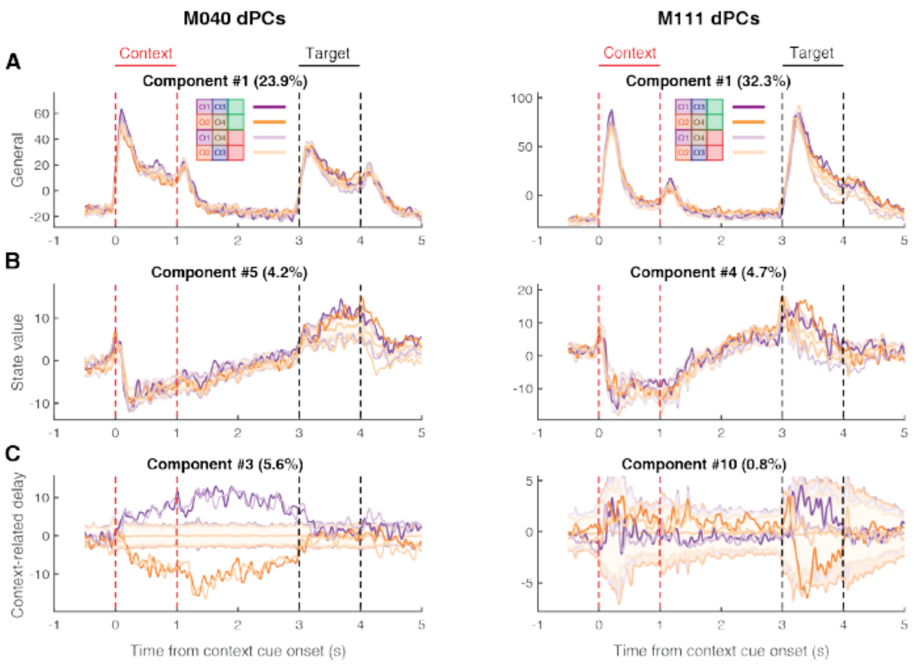

Note that in these panels (taken from Figure 7) the cue-general signal (A) and ramping signal (B) overlap with the shaded bootstrap area, indicating they are not dependent on context cue identity. In contrast, panel C shows a context cue-dependent component which is clearly outside the shaded area (for M040, left column, but not M111, right column).

[As an aside, to determine significance of the cue-independent components, a different shuffle would be more appropriate, such as temporally shifting the times of cue presentation. However, since this is not the focus of the paper, we do not include that analysis here.]

In response, we have updated the text in the following ways (changes in **bold**):

Results, p. 13 (lines 193-197):

“To test components that differ across trial types, we compared the components extracted from the data to components from a shuffled distribution extracted by shuffling the trial identity of each trial**, while leaving the within-trial temporal dynamics unaltered** (note, this process will preserve any general time-varying component that is independent of trial type).”

Updated Figure 7 legend (p. 16):

“Shaded regions and associated lines indicate 2.58 standard deviations ($p < 0.01$) and the mean of a shuffled distribution where trial identity was shuffled before trial-averaging the data. **This shuffling procedure removes information about trial identity while preserving the temporal dynamics of condition-invariant signals.”**

Added shuffling information in Methods (p. 31, lines 577-582):

“In our task, we sought to `demix' the contributions of the context cue, target cue, and their interaction (e.g. ``outcome-related'') across time, projecting the data using components derived from these task variables, and visualized how the projected neural trajectories evolved throughout a trial in this reduced dPCA space (see Figure 6B for hypothetical trajectories along context and outcome axes). **We then visualized the differences across trial types in these components via comparison to a shuffled distribution that shuffled the trial identity of each trial, while preserving the temporal dynamics, preserving condition-invariant signals.”**

The new figure S3 is a very useful addition. It does, however, raise a new question: there seem to be multiple components that match the expected patterns for some of the encoding types. For example, the authors chose component #3 of mouse M142 as the state value component, but to my eye component #4 looks even stronger (at least in terms of a ramping signal). The same applies to components 2 vs 3 for M146. It seems there was no objective criterion for choosing the component that *best* matched a particular pattern. In these two cases the authors chose the ones that explained the greater amount of variance, potentially biasing their conclusions

We agree with the reviewer that our selection of the “state value” components was not objective. To address this, we have implemented a linear regression predicting the condition-averaged projected signal for each component from the time between the context and target cues. Intuitively, if the striatal signal is ramping during this period, we should see a positive relationship between increases in time and the data. The results of this linear regression fit showed that each mouse had a single component that was well-described by such a linear fit with a positive slope (adjusted R-squared, M040; 0.90, M111: 0.93; M142: 0.91; M146: 0.79), which was substantial larger than the next closest component (next largest R-squared with a positive relationship, M040: 0.32; M111: 0.60; M142: 0.23; M146: 0.48). This included the same components originally reported for M040 and M111, and the components suggested by the reviewer for M142 and M146. We have updated the manuscript in the following ways:

Initial report of “state value” signals in the Results (p. 14, line 200):

“Another strong non-specific signal observed across mice was a ramping signal that increased in magnitude from context cue onset to after target cue onset (Figures 5B, 7B; variance explained, M040: 4.2%; M111: 4.7%; M142: 5.5%; M146: 4.5%). Furthermore, there was a significant positive linear relationship between this component and time (adjusted R-squared, M040: 0.90; M111: 0.93; M142: 0.91; M146: 0.79), that was substantial larger than the next closest component and time (next largest R-squared with a positive relationship M040: 032; M111: 0.60; M142: 0.23; M146: 0.48), consistent with what would be expected from a ramping ``state value'' signal.”

Updated accompanying components for M142 and M146 in Figure 7, as well as the figure legend:

“**B**: The extracted component from each mouse that best represents a state value signal, with a ramping-like activity between context cue offset and target cue onset. Note the variability in this component across mice after target cue onset, in particular the separation between rewarded (dark colors) and unrewarded (light colors) trials for M040 and M111.”

Updated relationship of this component for M142 and M146 with the delay context-related component (p. 16, line 214):

“The dot product between the delay context-related component and the general cue (M040: 0.07; $p = 0.136$; M111: 0.05; $p = 0.219$; M142: 0.18; $p < 0.001$; M146: 0.24; $p = 0.002$) and state value (M040: -0.07; $p = 0.156$; M111: 0.05; $p = 0.242$; **M142: 0.04; $p = 0.259$; M146: 0.26; $p < 0.001$**) components did not significantly deviate from zero in some, but not all, mice, suggesting that in some cases these signals are orthogonal and can coexist independently within the same population of NAc units.”

Updated Discussion (p. 26, line 428):

“For instance, all mice showed a ramping component after context cue onset, consistent with dynamics that closely mimic what would be expected from a signal conveying state value.”

Updated methods to add linear regression (p. 32, line 584):

“To identify the ramping ``state value`` component, we implemented a linear regression predicting the condition-averaged projected activity from each component with the time between context cue offset and target cue onset.”

Finally, we updated the labeling of these components in Figure S3.

Editorial comments:

1) Abstract: Please try to make your abstract more accessible to a general life science readership. You could ask a scientist in an unrelated field to read it.

Thank you for this important suggestion. We have removed several instances of jargon and technical language in the abstract, replacing it with more accessible concepts.

Original:

“Neural activity in the nucleus accumbens (NAc) is thought to track fundamentally value-centric quantities such as current or future expected reward, reward prediction errors, the value of work, opportunity cost, and approach vigor. However, the NAc also contributes to flexible behavior in ways that are difficult to explain based on value signals alone, raising the question of if and how non-value signals are encoded in NAc. We recorded NAc neural ensembles while head-fixed mice performed a biconditional discrimination task where context-setting cues modulated the stimulus-outcome association of subsequently presented reward-predictive cues. We extracted single-unit and population-level correlates of task features, and found value-independent coding for the context cues. This context signal occupied a subspace orthogonal to outcome-predictive representations, and was predictive of subsequent behaviorally-relevant coding. Together, these findings support a circuit-level gating model for how the NAc contributes to behavioral flexibility and provide a novel population-level perspective from which to view NAc computations.”

Edited:

“Neural activity in the nucleus accumbens (NAc) is thought to track fundamentally value-centric quantities **linked to reward and effort**. However, the NAc also contributes to flexible behavior in ways that are difficult to explain based on value signals alone, raising the question of if and how non-value signals are encoded in NAc. We recorded NAc neural ensembles while head-fixed mice performed an **odor-based** biconditional discrimination task where **an initial discrete cue** modulated the **behavioral significance** of **a** subsequently presented reward-predictive cue. We extracted single-unit and population-level correlates **related to the cues**, and found value-independent coding for the **initial, context-setting** cue. This context signal occupied a **population-level coding space** orthogonal to outcome-**related** representations, and was predictive of subsequent behaviorally-relevant responses to the

reward-predictive cues. Together, these findings support a **gating** model for how the NAc contributes to behavioral flexibility and provide a novel population-level perspective from which to view NAc computations."

2) Ethics:

2.1) Please include the ID number of your protocol(s) approved by the Dartmouth College Institutional Animal Care and Use Committee (IACUC).

2.2) Please include the specific national or international regulations/guidelines to which your animal care and use protocol adhered. Please note that institutional or accreditation organization guidelines (such as AAALAC) do not meet this requirement.

We have added the ID number of the protocol and the national guidelines in the methods section:

"All experimental procedures were approved by the Dartmouth College Institutional Animal Care and Use Committee (IACUC; **protocol #00002171), and carried out in accordance with the National Institute of Health's Guide for the Care and Use of Laboratory Animals."**

3) Data:

You may be aware of the PLOS Data Policy, which requires that all data be made available without restriction: http://journals.plos.org/plosbiology/s/data-availability. For more information, please also see this editorial: http://dx.doi.org/10.1371/journal.pbio.1001797

Note that we do not require all raw data. Rather, we ask for all individual quantitative observations that underlie the data summarized in the figures and results of your paper. For an example see here:
http://www.plosbiology.org/article/info%3Adoi%2F10.1371%2Fjournal.pbio.1001908#s5

3.1) We note that you mention that "All preprocessed data files are available on DataLad (http://datasets.datalad.org/, BiconditionalOdor data set)". However, we could not find the dataset. Could you please provide a more detailed explanation? Could you also include a README file that explains how the preprocessed data were analyzed to generate all quantitative plots and graphs in all your figures, including supporting figures?

Data was uploaded to the following repository on GIN:
https://gin.g-node.org/jgmaz/BiconditionalOdor.

3.2) Please also ensure that each figure legend in your manuscript includes information on where the underlying data can be found.

Data location was added to the end of each data-containing figure (e.g. Data: https://gin.g-node.org/jgmaz/BiconditionalOdor.)

3.3) Please ensure that your Data Statement in the submission system accurately describes where your data can be found.

Done
